# Relationship between the Systemic Immune- Inflammation Index and Muscle Strength in Patients with Chronic Kidney Disease: A Cross- Sectional Study of Data from NHANES 2011–2014

**DOI:** 10.2174/0118715303430103251204120642

**Published:** 2026-06-22

**Authors:** Qianqian Zhang, Zhen Jia, Huijuan Hou, Xiangdong Yang

**Affiliations:** 1 Department of Nephrology, Qilu Hospital of Shandong University, Jinan, 250000, China;; 2 Department of Nephrology, Qilu Hospital of Shandong University, Dezhou Hospital, Dezhou, 253000, China;; 3 Department of Cardiovascular, Qilu Hospital of Shandong University, Dezhou Hospital, Dezhou, 253000, China

**Keywords:** Systemic immune-inflammation index, low muscle strength, chronic kidney disease, NHANES, cross-sectional study, sarcopenia

## Abstract

**Introduction:**

This study aimed to determine the relationship between Systemic Immune-Inflammation Index (SII) and muscle strength in patients with CKD.

**Methods:**

This cross-sectional study examined adults with CKD using data from the National Health and Nutrition Examination Survey (2011–2014). The SII was calculated as: platelet count × neutrophil count/lymphocyte count. Muscle strength was determined from measurements of handgrip strength. Multivariate logistic regression and additional methods were used to assess the relationship between SII and muscle strength.

**Results:**

We examined 1620 patients with CKD (mean age: 61.4 years; 47% male). The overall prevalence of low muscle strength was 12.04%, and curve fitting indicated a positive, linear relationship between SII and low muscle strength. However, multivariate logistic regression analysis indicated that this relationship was not significant after adjustment for multiple confounders (odds ratio: 1.01, 95% CI: 0.99–1.02). These results were robust, and subgroup analyses showed no interactions.

**Discussion:**

Inflammation, malnutrition, and chronic comorbidities can affect muscle metabolism in CKD patients. The relationship between SII and low muscle strength is complex and variable. Although SII reflects systemic immune activation, it is less capable than markers, such as IL-6 and CRP, in directly capturing tissue-level inflammation that leads to muscle atrophy.

**Conclusion:**

There was no significant association between SII and low muscle strength in patients with CKD, even after adjustment for nutritional status, comorbidities, and renal function. Although previous studies showed that CKD patients had increased inflammatory responses and decreased muscle strength, our results suggest underlying complexities may confound this relationship.

## 1 INTRODUCTION

Sarcopenia, the slow and continuous loss of muscle mass and function due to age or physical disability, has become a major concern in clinical practice due to the aging population and the increasing prevalence of many chronic diseases, including chronic kidney disease (CKD) [1, 2]. Previous research reported that the prevalence of sarcopenia among patients with all stages of CKD was 4% to 42% [3]. Another study reported that the prevalence of severe sarcopenia among patients with CKD was 21.0%, the rate was greater for patients on dialysis, and low muscle strength was present in almost half of all patients with CKD [1].

Sarcopenia is a serious condition for many reasons, and low muscle strength is an important component of sarcopenia. Low muscle strength is associated with an increased risk of falls and fractures [4, 5]; it disrupts the ability to perform activities of daily living [6]; it is associated with an increased risk of cardiac disease [7], respiratory disease [8], and cognitive impairment [9]. Additionally, it can lead to permanent immobility [2] and decreased quality of life [10], dependence on others for care [11-13], and premature death [14]. Low muscle strength is also associated with significant healthcare costs.

A 2017 meta-analysis found that higher levels of multiple inflammation biomarkers were associated with decreased muscle strength and muscle mass [15]. Other recent studies have concluded that chronic systemic inflammatory processes play a crucial role in the progression of CKD-associated sarcopenia [16, 17]. Two studies on patients undergoing dialysis found that circulating inflammatory markers were higher in those with lower skeletal muscle strength and mass [18, 19]. Elevated levels of inflammatory cytokines can lead to decreased protein synthesis, reduced muscle anabolism, and disruption of the body’s energy balance, culminating in muscle atrophy and dysfunction [17]. Persistent systemic inflammation in patients with CKD, driven by elevated circulating pro-inflammatory cytokines, oxidative stress, metabolic acidosis, infections, impaired adipose metabolism, and decreased intestinal function, is positively associated with all-cause mortality [20].

Patients with CKD have higher circulating concentrations of C-reactive protein (CRP), interleukin-6 (IL-6), tumor necrosis factor alpha (TNF-α), and other cytokines. Development of the inflammatory state during CKD can lead to muscle atrophy because inflammatory cytokines activate the NF-κB pathway, which in turn stimulates muscle RING-finger protein-1 (MuRF1; an E3 ubiquitin ligase) and proteolysis by the ubiquitin-proteasome system (UPS). These changes also increase myostatin expression, which inhibits insulin-induced protein synthesis and signaling *via* the IGF-1/Akt pathway [21-24]. Systemic inflammation in CKD also disrupts hypothalamic function and responses to appetite-regulating hormones (leptin, ghrelin, and melanocortin), leading to persistent activation of anorexigenic neural pathways, decreased amino acid assimilation, decreased IGF-1 concentration, and impaired protein synthesis [25].

Previous researchers have used several tools to quantify systemic inflammation based on different routine biochemical or hematological indicators or ratios of different indicators [26]. The systemic immune-inflammatory index (SII) is calculated as: platelet count × neutrophil count/lymphocyte count [27, 28]. Previous studies found that the SII provided reliable assessments of local systemic inflammation and the body’s overall immunological response [29, 30]. These findings led to the use of the SII to predict prognosis in cancer patients. In particular, studies on the interaction of systemic inflammation and the local immune response considered this interaction as a hallmark of cancer because it affects the initiation and progression of many different cancers [31, 32], including cervical cancer [33], esophageal cancer [34], and hepatocellular carcinoma [35]. Moreover, Yang *et al*. studied patients with coronary artery disease (CAD) and found that the SII was associated with poor prognosis [36]. A 2023 cross-sectional survey of 10,367 American adults found that higher SII levels were associated with a higher risk of low muscle mass [37]. Another study found that inflammation and SII were positively associated with sarcopenia and sarcopenic obesity in older adults with chronic comorbidities. However, CRP was not significantly associated with these two conditions [38]. Zeng *et al*. found a significant relationship between SII and risk of mortality in a U.S. cohort of adults with sarcopenia [39]. These previous studies provide a rationale for studying the relationship of SII with muscle- related outcomes in patients with CKD.

Therefore, we conducted a population-based cross-sectional study using data from the National Health and Nutrition Examination Survey (NHANES) to investigate the relationship between the SII and low muscle strength in adults with CKD.

## 2 MATERIALS AND METHODS

### 2.1 Source of Data, Type of Study, and Study Design

This observational cross-sectional study used multivariate logistic regression and additional methods to determine the relationship between the SII and muscle strength in patients with CKD. All demographic and laboratory data were from NHANES (2011–2014) [40, 41], an ongoing study performed in the United States by the National Center for Health Statistics (NCHS) of the Centers for Disease Control and Prevention (CDC). The CDC Institutional Review Board approved the study protocol, and all participants provided written informed consent [42, 43]. All NHANES data were collected by trained professionals using at-home interviews and examinations [40, 44]. The study population was selected using complex, multistage, stratified, cluster probability sampling and was representative of the overall United States population. The authors had no access to information that could identify individual participants during or after data collection. As the present study was a secondary analysis, it did not require approval from a local institutional review board [45]. All data in the NHANES can be accessed at https://wwwn.cdc.gov/nchs/nhanes/default.aspx. Data downloaded on 1^st^ February, 2024, were used in the present study.

### 2.2 Body Composition, Handgrip Strength, and Definition of Low Muscle Strength

The height and weight of enrolled participants were recorded, and body mass index (BMI) was calculated as kg/m^2^. Handgrip strength (HGS) was determined during two cycles of NHANES (2011–2014) using a Takei Dynamometer (TKK 5401; Takei Scientific Instruments, Tokyo, Japan). In this test, each subject was asked to stand upright with the arms directed downward [46]. The test was then performed three times for each hand (dominant and nondominant), with 60 s between measurements. The greatest recorded strength for each hand was used for subsequent analysis. Multiple international groups use low HGS (females < 18 kg; males < 28 kg) as one of the criteria for defining sarcopenia [47].

### 2.3 SII and Other Covariates

The SII was defined as peripheral platelet count × neutrophil count/lymphocyte count. The following patient data were recorded: age; sex; race (non-Hispanic white, non-Hispanic black, Hispanic, or other); self-reported smoking status (never smoker: <100 lifetime cigarettes and does not smoke now; former smoker: >100 lifetime cigarettes and does not smoke now; current smoker: >100 lifetime cigarettes and smokes now); self-reported drinking status (yes: ≥12 lifetime alcohol drinks; no: <12 lifetime alcohol drinks); marital status (living alone: never married, separated, divorced, or widowed; married and living with partner); education (<9 years, 9–12 years, or >12 years); and family income based on the poverty income ratio (low: PIR ≤ 1.3, medium: PIR = 1.3–3.5, or high: PIR > 3.5) [48].

The presence of diabetes, hypertension, congestive heart failure, and stroke was based on self-reports. The laboratory data included: high-density lipoprotein (HDL), alanine aminotransferase (ALT), albumin (ALB), glycated hemoglobin (HbA1c), serum glucose (GLU), hemoglobin (Hb), blood urea nitrogen (BUN), white blood cells (WBCs), platelets (PLTs), lymphocytes (LYMs), and neutrophils (NEUs).

Multivariate analysis adjusted for the following potential covariates based on review of the literature [37, 38, 49]: age, sex, marital status, race/ethnicity, education level, family income, smoking status, physical activity, hypertension, diabetes, stroke, and coronary heart disease (CHD).

### 2.4 Chronic Kidney Disease

The estimated glomerular filtration rate (eGFR) was calculated using the 2021 CKD-EPI equation, which uses measurements of serum creatinine (SCr), age, and sex. CKD was defined as an eGFR below 60 mL/min/1.73 m^2^ or a urinary ALB-to-Cr ratio (UACR) of 30 mg/g or more [50].

### 2.5 Statistical Analysis

Qualitative variables are presented as n (%), and quantitative variables as mean (standard deviation, SD) or median (interquartile range, IQR), as appropriate. The significance of inter-group differences was determined using a one-way ANOVA (normally distributed quantitative variables), an independent-samples Kruskal-Wallis test (non-normally distributed quantitative variables), or the chi-square test (qualitative variables).

Logistic regression analysis, with calculations of odds ratios (ORs), adjusted ORs (aORs), and 95% confidence intervals (95%CIs), was used to determine the significance of the relationship between SII and low muscle strength. Four different multivariate models were used to correct for confounding by different variables. The relationship between SII and low muscle strength was also analyzed using smooth curve fitting of multivariate Model V with the restricted cubic spline (RCS) algorithm. The heterogeneity of SII subgroups and their interactions with the SII were determined using likelihood ratio testing and calculation of interaction *P*-values.

An *a priori* estimate of statistical power for the study population was not performed because the sample size was based on previously collected data. All statistical calculations were performed using the Free Statistics software version 1.9 [51]. A *P*-value below 0.05 in a two-tailed test was considered significant.

## 3 RESULTS

### 3.1 Study Population

A total of 19,931 participants in the NHANES (2011–2024) completed the interview, and 2572 were diagnosed with CKD (Fig. **1**). We excluded subjects aged < 20 years (n = 700), pregnant women (n = 13), subjects missing data on handgrip strength (n = 186), and subjects missing data needed to calculate the SII (n = 53). There were 1620 CKD patients in the final analysis. Among these patients, 195 (12.04%) had low muscle strength based on HGS measurements (female < 18 kg, male < 28 kg), whereas the other 1425 CKD participants did not.

### 3.2 Baseline Characteristics

We classified all 1620 CKD patients into four quartiles (405 per group) based on the SII value (Table **1**): Q1 (SII < 331.191), Q2 (331.191 ≤ SII < 490.350), Q3 (490.350 ≤ SII < 723.898), and Q4 (SII ≥ 723.898). Among all 1620 patients (mean age: 61.4 years, 47% males), the average SII was 589.749, and the median SII was 490.350. The prevalence of low muscle strength increased with SII (*P* = 0.029). In addition, the four groups also had significant differences in Hb, ALB, GLU, BMI, race/ethnicity, previous stroke, alcohol consumption, smoking, and UACR (all *P* < 0.05).

### 3.3 Relationship between SII and Low Muscle Strength: Univariate Analysis

We performed a univariate analysis to identify variables associated with low muscle strength (Table **2**). The results demonstrated that greater age, living alone, high SII, high urinary ACR, high BUN, high ALP, hypertension, diabetes, stroke, and CHD increased the risk of low muscle strength. Non-Hispanic Black race/ethnicity, more education, low BMI, low Hb, low ALB, low ALT, low AT, current smoking, and alcohol consumption decreased the risk of low muscle strength. In this analysis, SII was entered as a continuous variable, with an increment of 30.

### 3.4 Relationship between SII and Low Muscle Strength: Multivariate Analysis

We performed a multivariate analysis to assess the significance of the association between SII and low muscle strength across four models (Table **3**). SII had a marginally significant association with low muscle strength when adjusting for age, sex, race/ethnicity, marital status, education level, and PIR (Model II, *P* = 0.043) and when adjusting for all Model II variables in addition to use of tobacco and alcohol (Model III, *P* = 0.049). However, this association was not significant when adjusting for all Model III variables in addition to stroke, hypertension, diabetes, and CHD (Model IV, *P* = 0.115) and when adjusting for all Model IV variables in addition to UACR, Hb, ALT, ALB, BUN, and BMI (Model V, *P* = 0.335).

Moreover, after adjusting for all confounders (Model V), the results of smooth curve fitting to this multivariate model showed a positive, nearly linear relationship between SII and low muscle strength (Fig. **2**). However, all of the 95% CIs (shaded regions in this figure) overlapped with an OR of 1.0, emphasizing that this relationship was not significant in Model V.

### 3.5 Relationship between SII and Low Muscle Strength: Stratified Analysis

We performed a stratified analysis to assess the potential effects of different variables on the relationship between SII and low muscle strength (Fig. **3**). In this analysis, apart from the variable being analyzed, we performed statistical adjustment for all other variables (age, race/ethnicity, marital status, *etc*.). The results showed that the effects of smoking and eGFR were significant (both *P* for interaction < 0.05). However, this result should be interpreted with caution, given the multiple testing and the similar directionality of the associations. We found no significant interactions between low muscle strength and the SII across all other subgroups (all *P* for interaction > 0.05).

## 4 DISCUSSION

### 4.1 Complex Relationship between SII and Low Muscle Strength

This cross-sectional study on 1620 CKD patients demonstrated that a high SII had a significant association with low muscle strength when there was no adjustment for confounding (Model I), in a model that adjusted for age, sex, race/ethnicity, marital status, education, and PIR (Model II), and in a model that adjusted for all Model II variables plus smoking status and alcohol consumption (Model III). However, this relationship was not significant after adjustment for all Model III variables plus stroke, hypertension, and diabetes (Model IV), or after adjustment for all Model IV variables plus UACR, Hb, ALT, ALB, BUN, and BMI (Model V). Our stratified analysis showed that smoking and eGFR affected the relationship of the SII with low muscle strength. Chronic diseases, such as CKD, cancer, long-term infection, and aging, can induce an imbalance of protein synthesis and degradation in skeletal muscle, triggering muscle wasting and even atrophy. However, we found positive associations between SII and low muscle strength in subgroups with low eGFR and in never smokers (even though renal insufficiency can lead to chronic inflammation) [52], but negative associations in subgroups with normal eGFR and former smokers. This heterogeneity is biologically plausible and noteworthy, indicating that the relationship between SII and low muscle strength is complex, as several specific clinical variables alter its nature.

### 4.2 Cytokines, Inflammation, and Muscle Atrophy

The persistence of systemic inflammation in patients with CKD increases the risk of all-cause mortality. Many patient characteristics contribute to systemic inflammation, including levels of pro-inflammatory cytokines in the circulation, oxidative stress, metabolic acidosis, infections, altered fat metabolism, and intestinal dysfunction. Thus, complex interactions were found among cytokines, inflammation, and muscle atrophy [53]. It is important to consider that moderate levels of certain inflammatory cytokines are essential for normal muscle growth and development and for promoting muscle repair. In particular, TNF promotes muscle growth after injury [54]. In addition, patients with CKD exhibit increased IL-6 release from muscle tissue during hemodialysis, which can enhance muscle protein synthesis [55]. Conversely, other pro-inflammatory factors increase muscle atrophy in CKD [22].

### 4.3 SII Correlates with Various Pathological Conditions

The SII is a reliable marker of *in vivo* inflammation, and its level correlates with various pathological conditions. For example, a NHANES-based study used a two-segment linear regression model and reported a positive, but non-linear, association between SII and hyperlipidemia, with an SII inflection point at 479.15 (corresponding to our Q3 or Q4; Table **1**) [49]. Another study of a Chinese cohort of 218 patients with severe pancreatitis found that the SII level reliably predicted acute kidney injury [56]. A 2021 retrospective study reported that the SII at admission was a significant predictor of vasospasm [57]. A 2024 multi-center retrospective cohort study reported that the SII had a significant relationship with all-cause mortality in patients with sarcopenia and sarcopenic obesity who were undergoing peritoneal dialysis [58].

### 4.4 SII and Low Muscle Strength

Clinicians often use grip strength to assess low muscle strength, and the combination of low muscle strength and low muscle mass is used to diagnose sarcopenia. A 2022 cross-sectional study of 8232 individuals that used the NHANES database found that grip strength was a predictor of immune and inflammatory status [46]. Another study examining older adults with different chronic comorbidities reported that the SII showed positive correlations with low muscle mass and sarcopenic obesity, whereas CRP did not show significant correlations with either variable [38]. A 2023 study also reported that an increased SII in the general population was associated with low muscle mass [37]. A 2022 study of patients with CKD found that an inflammatory diet was independently associated with low muscle mass, and that an anti-inflammatory diet protected against CKD-related low muscle mass [59]. A Chinese study of 272 hemodialysis patients with sarcopenia found that the neutrophil/lymphocyte ratio had a significantly positive relationship with sarcopenia in the overweight group but not in the non-overweight group [60].

However, in contrast to other inflammatory markers, such as CRP, IL-6, and TNF-α, our results suggest that SII may not directly reflect the pathways that promote muscle loss in CKD. We can suggest two possible reasons for this. First, CKD patients, especially those with GFR below 60 mL/min/1.73 m^2^, already exhibit sustained inflammation, potentially masking the predictive value of SII. Second, low muscle strength is a complex condition, and muscle wasting in CKD is driven by many factors, including the accumulation of uremic toxins, metabolic acidosis, and oxidative stress. These factors may diminish the predictive value of SII, which is based only on counts of platelets, neutrophils, and lymphocytes. Previous studies reported associations between IL-6 and CRP and low muscle strength in CKD/hemodialysis patients [22, 61]. These two markers may more directly reflect muscle protein degradation and muscle atrophy, activation of the UPS pathway, or impaired satellite cell function. As a hematologic index, the SII might correlate with systemic immune activation, but not with the tissue-level inflammation that drives muscle atrophy.

It is noteworthy that activation of protein catabolism is a more prominent cause of muscle wasting than decreased protein synthesis in patients with CKD. These patients have elevated blood levels of pro-inflammatory cytokines (*e.g*., IL-6, TNF-α). Inflammatory stimuli (*e.g*., Angiotensin II) increase IL-6, which activates Signal Transducer and Activator of Transcription 3 (STAT3) in muscle tissues; STAT3 then upregulates the transcription factor C/EBPδ, which increases the expression of myostatin, a protein that inhibits muscle growth and promotes protein degradation. IL-6 induction of SOCS-3 interferes with insulin/IGF-1 signaling, thereby suppressing muscle synthesis and promoting muscle breakdown. Inflammatory cytokines (*e.g*., TNFα, IL-6, and IL-1) can also directly activate major protein degradation systems, including the UPS [62]. However, lymphocytes and neutrophils directly activate the STAT3/myostatin pathway and inhibit insulin signaling by releasing inflammatory factors (*e.g*., TNFα, IFNγ, and reactive oxygen species), thereby triggering protein degradation. Platelets amplify the inhibitory effect of myostatin by releasing mediators, such as TGFβ, and these alterations collectively exacerbate muscle atrophy in CKD. Therefore, before considering clinical variables related to nutrition (*e.g*., Hb, ALB, BMI), we found a positive relationship between SII and low muscle strength. Anemia and malnutrition are strong drivers of muscle weakness and may confound the effects of systemic inflammation as measured by SII [22, 63, 64].

### 4.5 Perspectives and Limitations

We believe that additional studies are needed to examine the mechanisms by which the individual components of SII, such as platelets and neutrophils, are associated with loss of skeletal muscle in CKD. Future studies should also assess the use of the SII to monitor the progression of low muscle strength over time while accounting for confounding by comorbidities, nutritional status, and clinical variables. SII should also be assessed alongside measurements of myokines, bone morphogenetic proteins (BMPs), and insulin-like growth factor-1 (IGF-1) to identify the most clinically relevant inflammatory or metabolic marker for low muscle strength in CKD patients. These studies may determine whether SII can predict CKD stage and examine the effects of dialysis modality and racial/ethnic group.

Our findings point to the complexities of inflammation in patients with CKD-related low muscle strength and highlight the importance of contextualizing inflammatory biomarkers within specific patient populations. Although our multivariable analysis (Models IV and V) demonstrated that the SII was not associated with muscle weakness in CKD patients, our results may help future researchers develop more precise hypotheses for studies examining loss of muscle strength in patients with CKD. Rigorous, multidisciplinary studies are essential to unravel these intricate interactions and guide evidence-based clinical interventions. We also need further research to determine whether the association between SII and low muscle strength observed in the present study also occurs in non-American CKD populations, particularly in Asian cohorts, where more extensive studies stratified by renal function levels are required.

The main novelty of the present study is that it was the first study to determine the relationship between low muscle strength and SII in a CKD population. The study had several strengths and weaknesses. We used a relatively large, representative sample of CKD patients from the United States, which allowed us to adjust for multiple confounders in four multivariable models. However, because our study was observational and cross-sectional, we can only examine the association between SII and low muscle strength and cannot infer causal relationships, particularly given the bidirectional relationship between inflammation and sarcopenia. Inflammation can increase muscle protein breakdown and decrease muscle strength by activating various pathways, such as the UPS and caspase-3 pathways. Patients with sarcopenia have abnormal metabolism of muscle and fat, and this can also lead to chronic inflammation in the body. NF-κB expression, which is linked to lipid metabolism deregulation, is relevant because it activates the NLRP3 inflammasome multiprotein complex, which initiates a network of immune responses associated with local and systemic inflammation [65].

It is also possible that our use of self-reported medical conditions may have been subject to recall bias. These limitations point to the need for prospective studies. Future studies on this topic should use a longitudinal design and include all these variables to investigate the mechanisms that connect inflammation and muscle metabolism. Furthermore, although our multivariable analyses adjusted for many confounders, we cannot exclude the effects of other unknown confounders. We did not stratify the analyses by CKD stage or dialysis status due to limited sample size. However, we acknowledge that the relationship between the studied variables may indeed differ by disease severity. The most widely cited definition of low muscle strength was proposed by the European Working Group on Sarcopenia in Older People (EWGSOP2); however, our definition of low muscle strength in the United States CKD population has not yet been validated. This is also a limitation of our research.

## CONCLUSION

Our cross-sectional study showed that a high SII was not significantly and independently associated with low muscle strength in a CKD population from NHANES. Therefore, our results raise questions about the nature of the SII's association with low muscle strength in patients with CKD.

## Figures and Tables

**Fig. (1):**
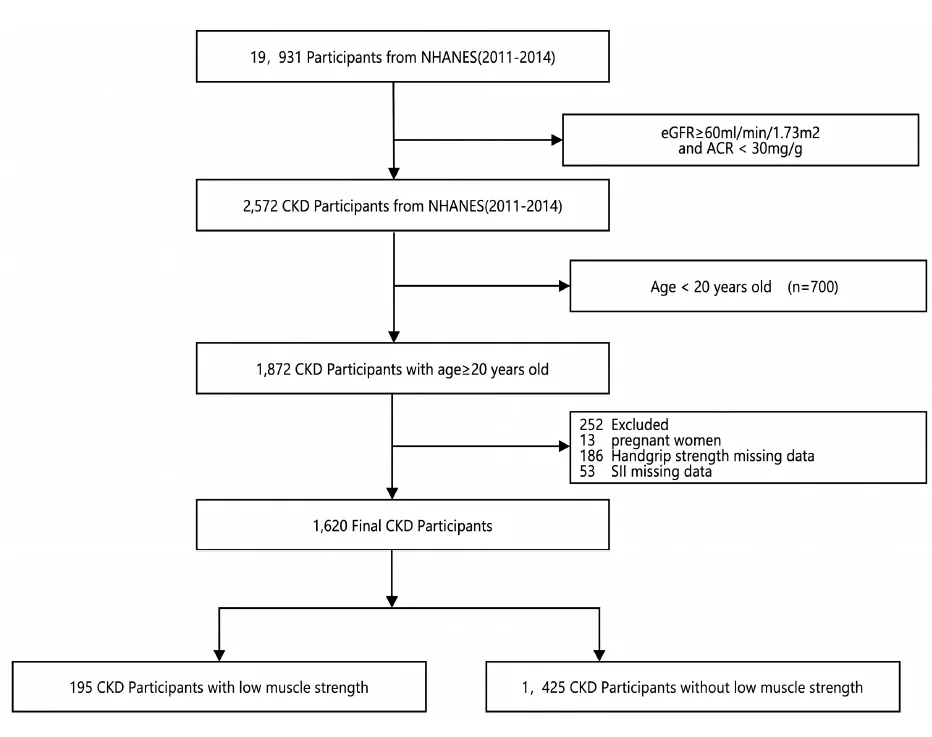
Disposition of 19,931 study subjects from NHANES (2011–2014) and final enrollment of 1620 CKD patients.

**Fig. (2):**
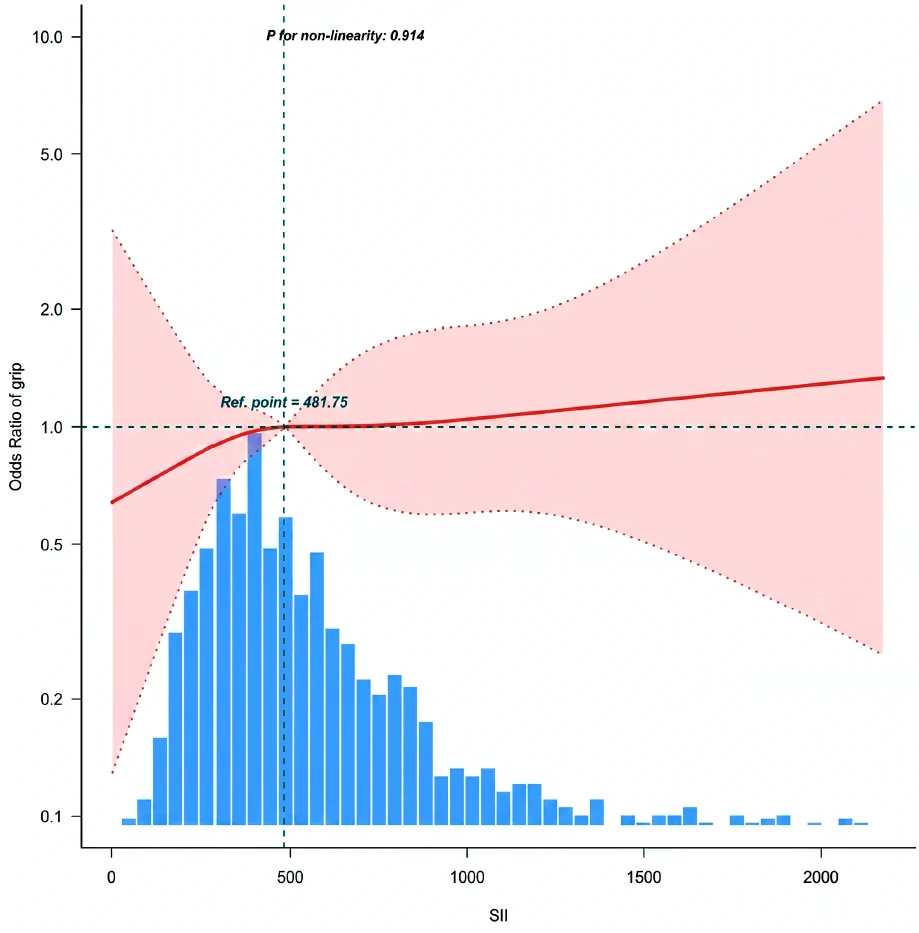
Smooth curve fitting of the relationship between SII and low muscle strength from the multivariate model V. Solid line: predicted aOR; shaded area: 95%CI; bars: binned data. Only 99% of the data is shown.

**Fig. (3):**
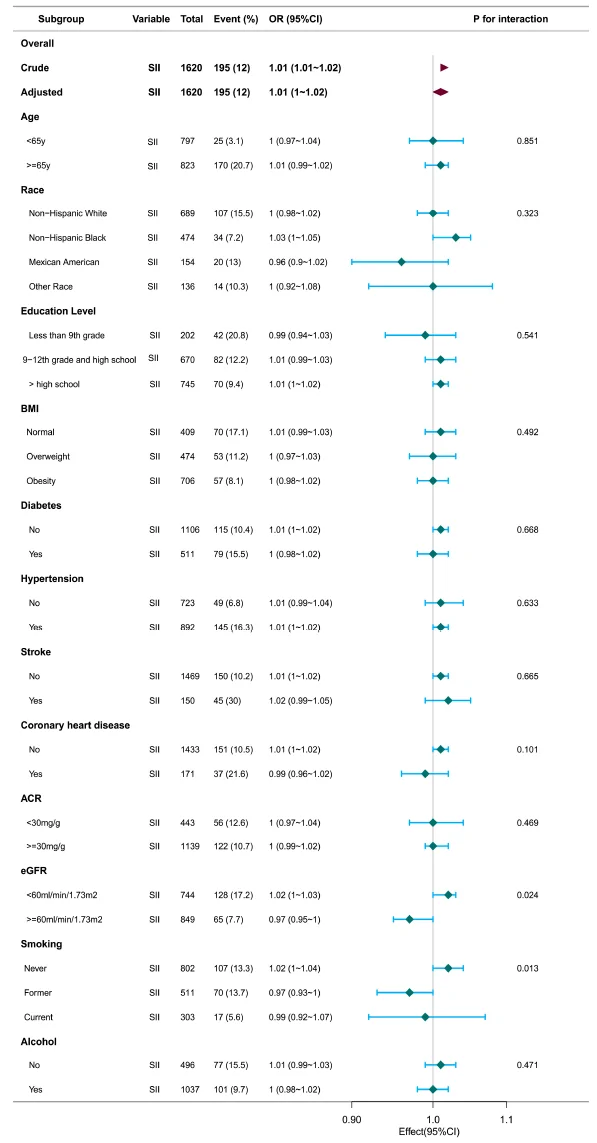
Stratified analysis of the relationship between SII and low muscle strength.

**Table 1:** Characteristics of all CKD patients and patients in different SII quartiles*.

Variable		Systemic Immune-Inflammation Index^†^						*P*-value
		All Patients	Q1		Q2	Q3	Q4	
Low Muscle Strength								0.029
No		1425 (88.0)	363 (89.6)		365 (90.1)	357 (88.1)	340 (84)	-
Yes		195 (12.0)	42 (10.4)		40 (9.9)	48 (11.9)	65 (16)	-
Age, years		61.4 ± 16.7	61.9 ± 16.2		60.6 ± 16.7	61.3 ± 17.2	61.9 ± 16.9	0.68
HDL (mmol/L)		1.3 ± 0.4	1.3 ± 0.4		1.3 ± 0.4	1.3 ± 0.4	1.3 ± 0.4	0.91
Hb (g/dL)		13.5 ± 1.7	13.5 ± 1.6		13.7 ± 1.6	13.5 ± 1.6	13.3 ± 1.8	0.025
Sex								0.471
Male		761 (47.0)	203 (50.1)	184 (45.4)		191 (47.2)	183 (45.2)	-
Female		859 (53.0)	202 (49.9)	221 (54.6)		214 (52.8)	222 (54.8)	-
BUN (mmol/L)		6.3 ± 3.4	6.2 ± 3.1	6.1 ± 2.9		6.4 ± 3.4	6.6 ± 4.0	0.097
ALB (g/L)		41.6 ± 3.4	41.9 ± 3.3	41.8 ± 3.1		41.3 ± 3.5	41.2 ± 3.8	0.009
GLU (mmol/L)		6.7 ± 3.3	6.5 ± 3.0	6.7 ± 3.4		6.6 ± 3.0	7.2 ± 3.9	0.008
BMI (kg/m^2^)		30.0 ± 7.4	29.1 ± 6.6	29.9 ± 7.2		30.2 ± 7.3	30.9 ± 8.3	0.006
ALT (U/L)		23.7 ± 37.1	23.4 ± 15.4	23.4 ± 17.8		21.8 ± 13.6	26.4 ± 69.0	0.377
eGFR (mL/min/1.73 m^2^)		71.6 ± 29.2	70.2 ± 28.1	73.1 ± 27.7		72.3 ± 30.5	70.9 ± 30.6	0.506
Marital Status								0.11
Married or living with a partner		829 (51.2)	212 (52.3)	222 (54.8)		207 (51.1)	188 (46.4)	-
Living alone		791 (48.8)	193 (47.7)	183 (45.2)		198 (48.9)	217 (53.6)	-
Race/Ethnicity								<0.001
Non-Hispanic White		689 (47.4)	129 (36.1)	165 (45)		184 (49.7)	211 (58.8)	-
Non-Hispanic Black		474 (32.6)	170 (47.6)	121 (33)		114 (30.8)	69 (19.2)	-
Mexican American		154 (10.6)	27 (7.6)	42 (11.4)		37 (10)	48 (13.4)	-
Other		136 (9.4)	31 (8.7)	39 (10.6)		35 (9.5)	31 (8.6)	-
Education Level								0.183
Less than grade 9		202 (12.5)	54 (13.4)	60 (14.9)		41 (10.1)	47 (11.6)	-
Grade 9–12 and High School		670 (41.4)	177 (43.8)	158 (39.1)		179 (44.2)	156 (38.6)	-
>High School		745 (46.1)	173 (42.8)	186 (46)		185 (45.7)	201 (49.8)	-
Stroke								0.002
No		1469 (90.7)	377 (93.3)	379 (93.6)		360 (88.9)	353 (87.2)	-
Yes		150 (9.3)	27 (6.7)	26 (6.4)		45 (11.1)	52 (12.8)	-
Coronary Heart Disease								0.332
No		1433 (89.3)	358 (88.8)	365 (91)		360 (90.2)	350 (87.3)	-
Yes		171 (10.7)	45 (11.2)	36 (9)		39 (9.8)	51 (12.7)	-
Hypertension								0.138
No		723 (44.8)	187 (46.3)	187 (46.4)		188 (46.7)	161 (39.8)	-
Yes		892 (55.2)	217 (53.7)	216 (53.6)		215 (53.3)	244 (60.2)	-
Alcohol Consumption								0.036
No	496 (32.4)		146 (38.3)	121 (31.3)		112 (29.6)	117 (30.2)	-
Yes	1037 (67.6)		235 (61.7)	265 (68.7)		267 (70.4)	270 (69.8)	-
Diabetes								0.181
No	1106 (68.4)		289 (71.4)	281 (69.4)		276 (68.5)	260 (64.4)	-
Yes	511 (31.6)		116 (28.6)	124 (30.6)		127 (31.5)	144 (35.6)	-
Smoking Status								0.005
Never	802 (49.6)		216 (53.3)	212 (52.6)		199 (49.3)	175 (43.3)	-
Former	511 (31.6)		121 (29.9)	104 (25.8)		131 (32.4)	155 (38.4)	-
Current	303 (18.8)		68 (16.8)	87 (21.6)		74 (18.3)	74 (18.3)	-
Family Poverty Income Ratio								0.15
1.3–3.5	1166 (77.9)		305 (80.1)		281 (75.9)	281 (74.9)	299 (80.6)	-
>3.5	331 (22.1)		76 (19.9)		89 (24.1)	94 (25.1)	72 (19.4)	-
Urinary ACR	46.5 (21.4,108.2)		45.4 (15.3,105.5)		43.3 (20.2,101.6)	45.8 (23.1,101.9)	52.5 (30.6,130.2)	0.022

**Note:** * Values indicate n (%), mean ± SD, or median (IQR). ^†^Q1: SII < 331.191; Q2: 331.191 ≤ SII < 490.350; Q3: 490.350 ≤ SII < 723.898; Q4: SII ≥ 723.898.

**Table 2:** Univariate regression analysis of variables associated with low muscle strength.

Variable	OR (95%CI)	*P*-value
Age	1.11 (1.09~1.13)	<0.001
Sex (Female *vs.* Male)	0.99 (0.73~1.34)	0.951
Race/Ethnicity (Ref: non-Hispanic White)	-	-
Non-Hispanic Black	0.42 (0.28~0.63)	<0.001
Mexican American	0.81 (0.49~1.36)	0.426
Other	0.62 (0.35~1.13)	0.118
Education Level (Ref: less than grade 9)	-	-
Grade 9–12 and high school	0.53 (0.35~0.8)	0.003
>High school	0.4 (0.26~0.6)	<0.001
Marital Status (Ref: married or living with a partner)	-	-
Living alone	1.71 (1.26~2.32)	0.001
Family Poverty Income Ratio (Ref: 1.3-3.5)	-	-
>3.5	0.67 (0.44~1.01)	0.057
BMI	0.93 (0.91~0.96)	<0.001
SII*	1.01(1.01~1.02)	0.001
Urinary ACR	1 (1~1)	0.03
Hb	0.76 (0.69~0.83)	<0.001
GLU	1.01 (0.97~1.05)	0.718
ALB	0.89 (0.85~0.93)	<0.001
BUN	1.16 (1.11~1.2)	<0.001
ALT	0.96 (0.94~0.98)	<0.001
ALP	1 (1~1.01)	0.089
Smoking (Ref: Never)	-	-
Former	1.03 (0.75~1.43)	0.853
Current	0.39 (0.23~0.66)	<0.001
Alcohol consumption (Yes *vs.* No)	0.59 (0.43~0.81)	0.001
Hypertension (Yes *vs.* No)	2.67 (1.9~3.75)	<0.001
Diabetes (Yes *vs.* No)	1.58 (1.16~2.14)	0.004
Stroke (Yes *vs.* No)	3.77 (2.56~5.55)	<0.001
Coronary Heart Disease (Yes *vs.* No)	2.34 (1.57~3.5)	<0.001

**Note:** * SII was entered as a continuous variable, and the increment was 30.

**Table 3:** Multivariate regression analysis of the association of SII with low muscle strength in four different models.

SII*	OR (95%CI)	*P*-value
Model I (unadjusted)	1.01 (1.01~1.02)	0.001
Model II	1.01 (1~1.02)	0.043
Model III	1.01 (1~1.02)	0.049
Model IV	1.01 (1~1.02)	0.115
Model V	1.01 (0.99~1.02)	0.335

**Note:** * SII was entered as a continuous variable, and the increment was 30. Model I: unadjusted. Model II: adjusted for age + sex + race/ethnicity + marital status + education + PIR. Model III: Model II + smoking status + alcohol consumption. Model IV: Model III + stroke + hypertension + diabetes + coronary heart disease. Model V: Model IV + UACR + Hb + ALT + ALB + BUN + BMI.

## Data Availability

All data generated or analyzed during this study are included in this published article.

## LIST OF ABBREVIATIONS

CKD: Chronic Kidney Disease
CRP: C-Reactive Protein
UPS: Ubiquitin-Proteasome System
SII: Systemic Immune-Inflammatory Index
CAD: Coronary Artery Disease
NHANES: National Health and Nutrition Examination Survey
NCHS: National Center for Health Statistics
BMI: Body Mass Index
HDL: High-Density Lipoprotein

## STANDARDS OF REPORTING

STROBE guidelines were followed.
